# Prevalence and genotype distribution of human papillomavirus among 9945 women from the Nanhai area of Foshan

**DOI:** 10.1186/s12879-019-3687-y

**Published:** 2019-01-18

**Authors:** Xiao-wen Yuan, Yi-Jian Li, Quan Qiu, Zi-yuan Luo, Xue-feng Zhao

**Affiliations:** Department of Clinical Laboratory, affiliated Nanhai Hospital of Southern Medical University, Foshan, 528200 Guangdong China

**Keywords:** Human papillomavirus, Genotype, Cervical carcinoma

## Abstract

**Background:**

To provide a scientific basis for the prevention and treatment of cervical cancer (CC), we investigated the distribution characteristics and genotypes of human papillomavirus (HPV) and the prevalence of multiple HPV infections in women of different ages seeking management for abnormal cytology in Foshan City.

**Methods:**

Screening for the 21 genotypes of HPV was carried out in 9945 females seeking management of abnormal cervical cytology results using rapid flow-through hybridization of nucleic acid molecules. The overall prevalence, genotype distribution and age-specific prevalence were examined.

**Results:**

Our results indicate that the prevalence of overall, high-risk, intermediate-risk, and low-risk HPV infections was 13.5%, 12.1%, 1.3%, and 1.9%, respectively. Of the 1346 women who tested positive, 89.5% were positive for a single HPV genotype, and 10.5% were positive for ≥2 genotypes. The most frequently detected HPV genotype was HPV-16 (2.9%), followed by HPV-52 (2.9%), HPV-58 (1.5%), and HPV-CP8304 (1.0%). The highest infection prevalence was found in patients 21–30 years old (271/1670, 16.2%).

**Conclusion:**

The prevalence of HPV infection in women seeking management for abnormal cytology in Foshan City is highest in the younger population (21–30 years old). Similar to most previous surveys, HPV-58 and HPV-52 infections are as common as HPV-16 infection.

## Background

Cervical cancer (CC) is the third most common cancer among females around the word [1], with more than 85% of cases occurring in developing countries, including China [2]. In 2012, 527,600 new cases of CC were diagnosed worldwide, with 265,700 women dying from the disease [3]. The development of preinvasive and invasive carcinogenesis requires persistent infection of high-risk human papillomavirus [4, 5], and CC screening can prevent a large proportion of cases. HPV genotypes are classified based on their oncogenic potential into high-risk subtypes (HPV-16, − 18, − 31, − 33, − 35, − 39, − 45, − 51, − 52, − 56, − 58, − 59, and − 68), intermediate-risk subtypes (66 and 53), or low-risk subtypes (HPV-6, − 11, − 42, − 43, − 44, and -CP8304) [6]. As the prevalence of HPV subtypes varies among regions and populations [7], HPV screening contributes to our understanding of the regional prevalence of HPV genotypes and its distribution, which aids in the design of HPV vaccines for the prevention of CC.

Here, we investigate cervical HPV infections among women seeking management of abnormal screening results to determine the age-specific and genotype-specific prevalence of HPV in the Nanhai area of Foshan.

## Methods

### Study population and enrollment

From January 2013 to July 2017, 9945 women who visited gynecologists or oncologists at affiliated Nanhai Hospital of Southern Medical University to seek management of abnormal cervical cytology results were enrolled; all patients provided informed consent. Inclusion criteria were as follows: (1) mentally and physically healthy; (2) age over 20 years; (3) permanent residency in the Nanhai area; (4) had participated in sexual intercourse at least once; (5) not pregnant or menstruating at the time of enrolment; (6) no history of total hysterectomy; (7) no history of cervical surgery; and (8) willingness to be tested for HPV genotypes.

### Specimen collection and DNA extraction

Physicians used a swab to swipe the region of the uterine cervix to ensure that cervico-vaginal cells were obtained. The collected samples were placed separately into vials containing 3 ml of preservation solution (Hybribio, Chaozhou, China) and stored at 4 °C until they were transported to the laboratory of Nan Hai Hospital affiliated with Southern Medical University, China, within 2 weeks for HPV DNA detection and typing. DNA extraction was conducted according to the MagPure Fast Blood DNA KF Kit instruction manual (Magen, Guangzhou, China).

### HPV genotyping

HPV detection and genotype analysis were performed using the HPV GenoArray Test Kit for 21 Types (Hybribio, Chaozhou, China); this chip technology can identify 21 different HPV genotypes, including 13 high-risk subtypes (HPV-16, − 18, − 31, − 33, − 35, − 39, − 45, − 51, − 52, − 56, − 58, − 59, and − 68), 2 intermediate-risk subtypes (66 and 53), and 6 low-risk subtypes (HPV-6, − 11, − 42, − 43, − 44, and -CP8304). In brief, 1 μl of sample DNA was mixed with 23.25 μl of PCR Mix and 0.75 μl of DNA Taq polymerase (5 U/μl), as provided in the kit. Both positive and negative controls supplied in the kit were included in every PCR test. Amplification was performed using an ABI VeritiDx PCR system (Applied Biosystems, USA). The PCR conditions were as follows: initial denaturation at 95 °C for 9 min, followed by 40 cycles of denaturation at 95 °C for 20 s, annealing at 55 °C for 30 s and extension at 72 °C for 30 s, with a final extension at 72 °C for 5 min. Amplicons were stored at 4 °C until denaturation, which was carried out in the thermal cycler at 95 °C for 5 min. The denatured product was applied onto the nylon membrane that contained immobilized HPV probes (HybriMem). The hybridization process was performed according to the manufacturer’s protocol. The assay utilized Hybribio’s proprietary Flow-through Hybridization Technology (US Patents 5,741,647 and 6,020,187), which actively directs targeting molecules toward the immobilized probes within the membrane fibers, with the complementary molecules being retained by the formation of duplexes. After a stringent wash, hybridization was detected by the addition of the streptavidin horseradish peroxidase conjugate, which binds to biotinylated PCR products and cleaves a substrate (nitro-blue tetrazolium-5-bromo-4-chloro- 3-indolylphosphate) to generate a blue-purple precipitate at the probe dot. Multiple-type infection was defined as one that was positive for at least 2 subtypes.

### Low limit of detection of the assay

The detection limit of the assay is 500 copies per reaction.

### Statistical analysis

All statistical analyses were performed using Excel and SPSS 19.0. Prevalence and 95% confidence intervals (CIs) were computed for any HPV infection, any high-risk type, any low-risk type, single- and multiple-type HPV infection, and individual genotypes. Data were stratified by age (21–30 years old, 31–40 years old, 41–50 years old, 51–60 years old, and > 60 years old). The Pearson Chi-squared test was used to assess the statistical significance of any differences in prevalence across age groups. *P* values < 0.05 were considered significant.

## Results

### Distribution characteristics of 9945 tested women

**Fig. 1:**
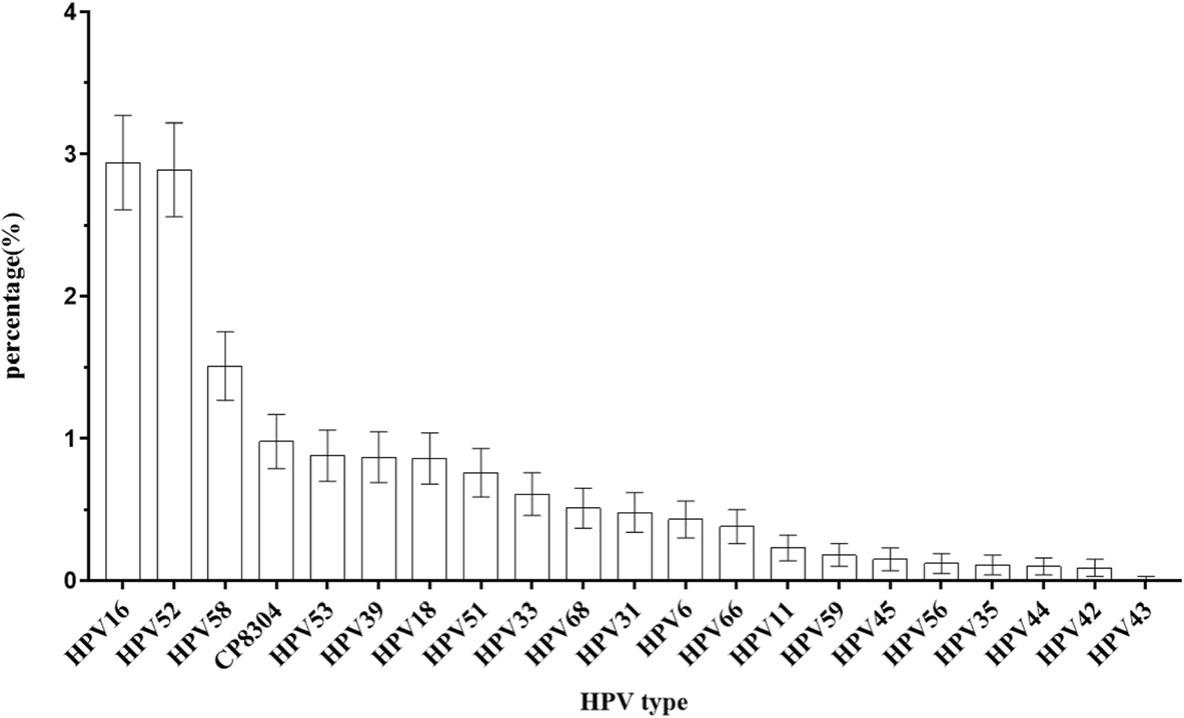
Prevalence and Distribution of HPV types among 9945 women from the Nanhai area of Foshan

A total of 9945 individuals (range, 21–90 years old) were included in the study. Genotype screening of the study participants indicated that the prevalence of overall, high-risk, intermediate-risk, and low-risk HPV infection was 13.5% (1346/9945, 95% CI 12.9% to 14.2)%, 10.6 (1057/9945, 95% CI 10.0 to 11.2%), 1.2% (116/9945, 95% CI 1.0 to 1.4%) and 1.7% (173/9945, 95% CI 1.5 to 2.0%), respectively. As shown in Fig. 1, the most frequently detected HPV genotype among women seeking management for abnormal cytology in Foshan City was HPV-16 (2.9, 95% CI 2.6 to 3.3%), followed by HPV-52 (2.9, 95% CI 2.6 to 3.2%), HPV-58 (1.5, 95% CI 1.3 to 1.7%), and HPV-CP8304 (1.0, 95% CI 0.8 to 1.2%.)

Of the 1346 women positive for HPV, 89.5% (1205/1346) were positive for a single HPV type, and 10.5% (141/1346) were positive for ≥2 types. Of those carrying more than one type, 87.9% (124/141) were infected with two genotypes, 9.9% (14/141) with three, and 2.8% (4/141) with four or more. Of the women infected by multiple HPV genotypes, 1 was infected by multiple low-risk (low-low) HPV genotypes, accounting for 0.7% of the patients with multiple HPV genotype infections, 0.1% of the HPV-positive cases, and 0.0% of all cases. In addition, 7 were infected by a combination of multiple high- and intermediate-risk subtypes (high-intermediate) HPV genotypes, accounting for 5.0% of the patients with multiple HPV genotype infections, 0.5% of the HPV-positive cases, and 0.1% of all cases; 122 were infected by multiple high-risk (high-high) HPV genotypes, accounting for 86.5% of the multiple HPV genotype infections, 9.1% of the HPV-positive cases, and 1.2% of all cases.

### Age-specific prevalence of HPV infection among the 9945 women

**Fig. 2:**
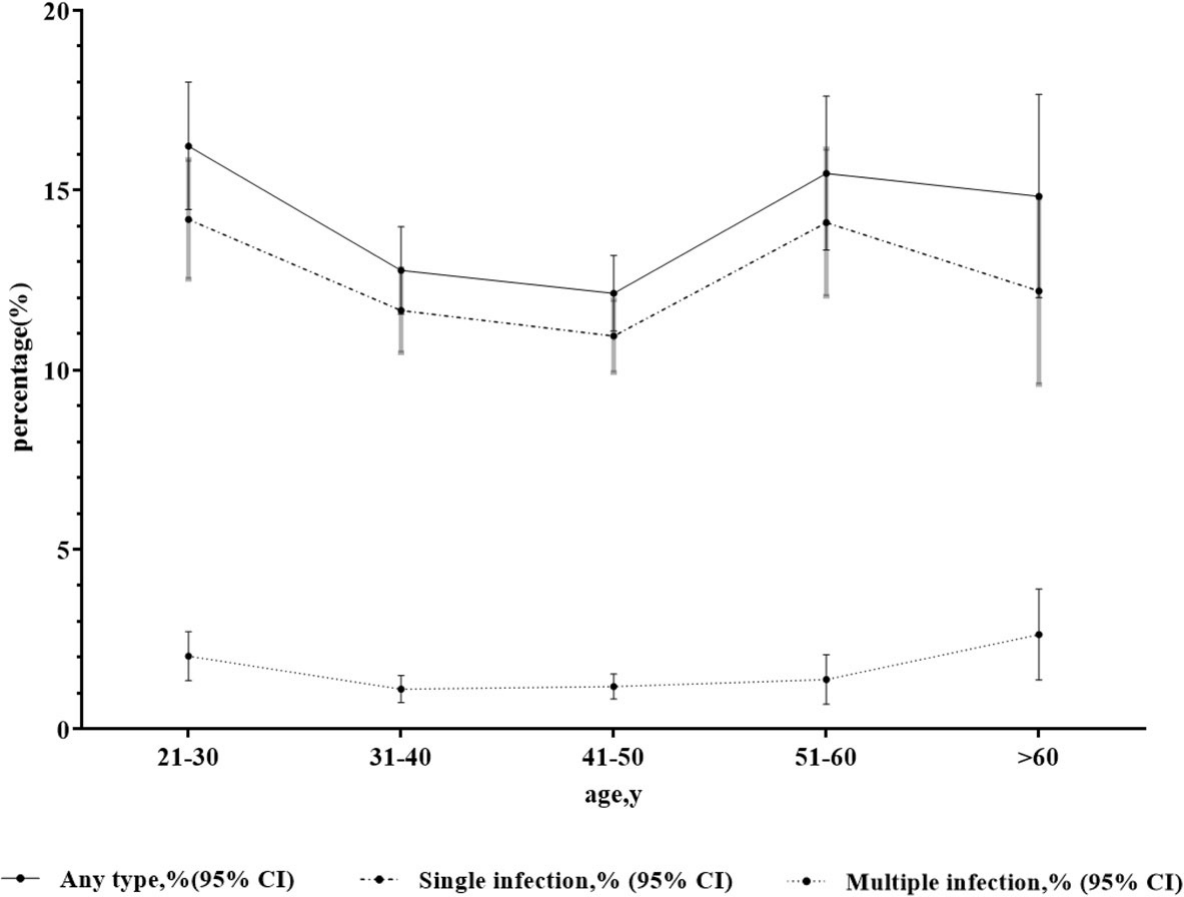
Age-specific prevalence of HPV infection. HPV: human papillomavirus. Multiple infection was defined as one that was positive for at least 2 types; CI: confidence interval

**Fig. 3:**
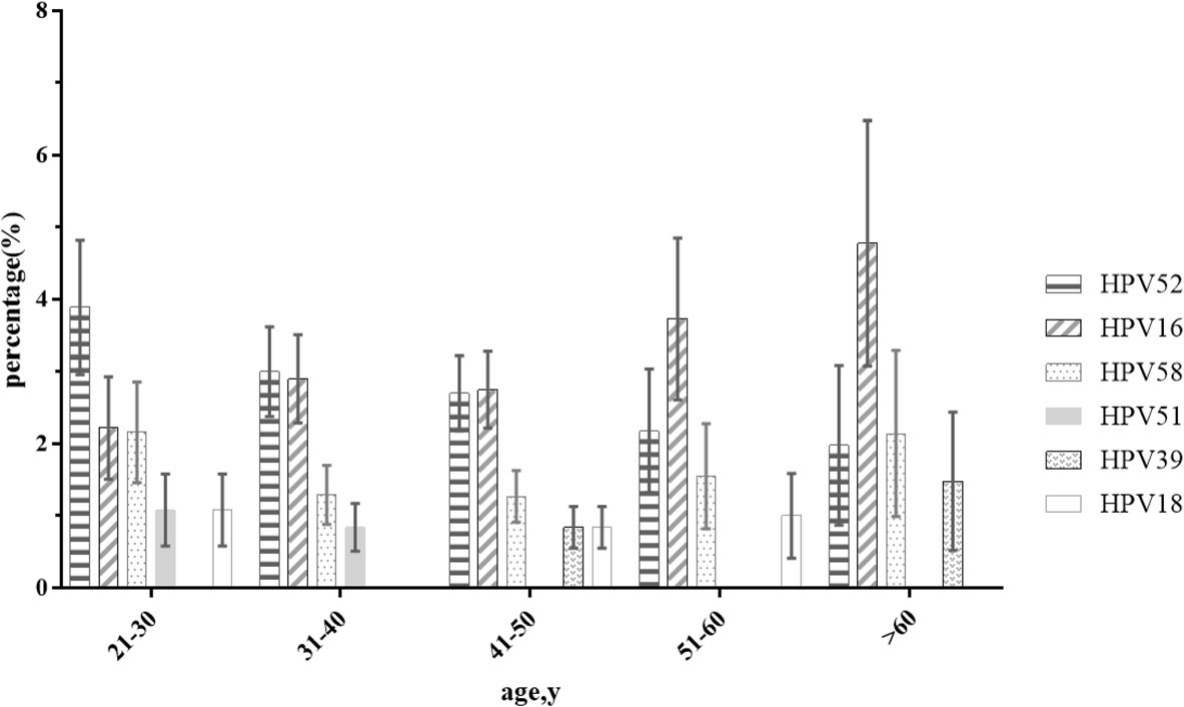
The most common HR-HPV genotypes among different age ranges. HPV: human papillomavirus. HR: high-risk

The women were divided into five age groups: 21–30 years old, 31–40 years old, 41–50 years old, 51–60 years old, and > 60 years old. Overall, HPV prevalence in women 21–30 years old, 31–40 years old, 41–50 years old, 51–60 years old, and > 60 years old was 16.2% (271/1670, 95% CI 14.5 to 18.0%), 12.8%(366/2866, 95% CI 11.5 to 14.0%), 12.1% (449/3703, 95% CI 11.1 to 13.2%), 15.5% (170/1099, 95% CI 13.3 to 17.6%) and 14.8% (90/607, 95% CI 12.0 to 17.7%), respectively. As shown in Fig. 2, there were two peaks of HPV infection based on age: the first was for women aged 21–30 years old and the second was for women aged 51–60 years old. Similar patterns of two peaks were also found for single infections, with the first peaks for multiple infection for women aged > 60 years old, of whom 2.6% were infected. While variations in prevalence by age were fairly minor (with differences of 4.1, 3.3, and 1.4% between the age groups with the highest and lowest prevalence for any HPV infection, single-type HPV infection, and multiple-type infection, respectively, differences across age groups were statistically significant (χ^2^ = 22.4, *p* = 0.001 for any HPV; χ^2^ = 16.2, *p* = 0.003 for single-type infection; χ^2^ = 14.3, *p* = 0.006 for multiple-type infection). The prevalence of HPV 16 and HPV 52 also differed among the age groups. As shown in Fig. 3, HPV-16、HPV-52 and HPV-58 were most frequently detected HPV genotype among the age groups.

## Discussion

Most HPV infections are transient and resolve spontaneously. However, CC generally develops with persistent high-risk HPV infection [4, 5]. Thus, a large proportion of cases can be prevented by screening. Data on the prevalence and genotype distribution of HPV infection in the Nanhai area of Foshan are incomplete. In this study, we investigated the profiles of HPV infection in women seeking management for abnormal cytology in Foshan city. Compared to similar studies of women seeking management for abnormal cervical cytology results in southeast China, the overall HPV prevalence (13.5%) in our study was different from that reported in Shanghai (31.8%) [8] and Chaozhou (24.5%) [9]. Moreover, compared with similar studies of women seeking management for abnormal cervical cytology results in Guangdong, the prevalence of high-risk HPV (12.1%) was lower than that reported in Chaozhou (19.5%) [9]. The distribution of HPV genotypes differed. According to population-based studies, the most common high-risk HPV subtypes in the overall female population worldwide are HPV-16, − 18, − 31, − 58, − 52, − 51 and − 33 [10]. Contrasting to most previous surveys in south of China [11–13] and other population [14], HPV-58 and HPV-52 infections are as common as HPV-16 infection.

For example, the most frequently detected HPV genotype among women in Zhejiang province [13] was HPV-52 (3.7%), followed by HPV-58 (2.7%) and HPV-16 (1.7%). And in Japan [14], HPV-52 (2.3%), HPV-16 (2.0%) and HPV-58 (1.6%) were the most frequently HPV genotypes. A similar prevalence pattern was also exhibited in our sample population; HPV-16 (2.9%) was the most commonly identified high-risk HPV subtype, followed by HPV-52 (2.9%) and-58 (1.5%). However, certain limitations must be considered when comparing our results to those from population-based studies. The limitation was that women seeking management of abnormal cervical cytology results are more likely to be HPV-positive than women in the general population. Our results showed that the three most prevalent high-risk genotypes were HPV-16, − 52 and − 58 and that HPV-CP8304 had the highest frequency among low-risk HPV genotypes in the Nanhai area of Foshan, which was similar to the previous study in Shanghai [8]. In our study, multiple HPV infections were identified in 10.5% (141/1346) of the HPV-infected women and in 1.4% of all specimens. High-risk HPV infections (involving high-high-risk and high-intermediate-risk) accounted for 9.4% (127/1346) of the positive specimens and 1.3% of all specimens, though only four patients were infected by four or more HPV genotypes.

Moreover, the distribution of HPV genotypes and their prevalence exhibited age-related differences. The highest overall prevalence of HPV was found in women 21–30 years old (271/1670, 16.2%), and the lowest HPV prevalence was found in women 41–50 years old (449/3703, 12.1%). The main reason for these findings may be the natural history of HPV infection and that the probability of acquisition cervical HPV infection is highest in young sexually active individuals and declines with age [15]. The highest prevalence of multiple HPV infections was found in women > 60 years old (16/90, 17.7%). According to population-based studies in America and Europe [10], age-specific prevalence curves were uneven. According to population-based studies in Hong Kong [12], variations in prevalence by age were quite uneven (with differences of 9.1 and 6.8% between the age groups with the highest and lowest prevalence for any HPV infection and single-type HPV infection). According to population-based studies in Zhejiang province [13], HPV infection prevalence in age group with the highest prevalence was 6.6% higher than those in age group with the lowest prevalence.

Moreover, according to similar studies of women seeking management for abnormal cervical cytology results in Shanghai [8] and Chaozhou [9], variations in prevalence by age were also quite uneven (with differences of 8.2 and 11% between the age groups with the highest and lowest prevalence for any HPV infection, respectively). However, there is little age-specific variation in prevalence, which is quite different from most population-based studies. Age-specific prevalence curves in our study were rather flat, even though differences across age groups were statistically significant due to the large sample size. This is probably due to the fact that the women were being seen for abnormal cytology, so there is little variation by age.

There are several limitations of the data in the present study. First, the prevalence data from the age groups over 60 are unreliable due to the fewer number of samples than the total number considered from the other age groups. Second, our study specifically recruited women seeking management for abnormal cervical cytology results and was not population-based. Therefore, our results only reflect the prevalence of HPV of women in Foshan City seeking management for abnormal cytology. In addition, we did not collect lifestyle information from every patient and did not investigate a detailed disease report for the participants. In the future, we will study the connection between lifestyle and HPV infection.

## Conclusions

In summary, our results suggest that the prevalence of HPV infection varies in women seeking management for abnormal cytology of different age groups. The prevalence of HPV infection in women seeking management for abnormal cytology is highest in the younger age group (21–30 years old). Moreover, HPV-16, − 52, − 58 and -CP8304 are the most common HPV subtypes among women seeking management of abnormal screening results in this region.

## Abbreviations

CC: Cervical cancer
CI: confidence interval
HPV: Human papillomavirus
hr-: High-risk
lr-: Low-risk
